# Pesticides and SLE: Is the Link Estrogenic?

**Published:** 2005-03

**Authors:** David J. Tenenbaum

Autoimmune diseases are multifactorial in nature and likely involve both environmental and genetic components. Estrogen is one environmental component that has been studied in relation to systemic lupus erythematosus (SLE) based on a number of clues. Like many other autoimmune diseases, SLE—a chronic disorder of uncertain cause and a varied clinical course—is more common among women, and onset is especially likely during childbearing years. In the (NZB × NZW)F_1_ mouse model of SLE, females develop more severe disease and die earlier than males; treating females with androgens (male hormones) slows the disease, while castrating males accelerates it. Several organochlorine pesticides can emulate estrogen in the body. Now a group of Florida researchers has assessed the possible role of these compounds in causing SLE **[*****EHP*** **113:323–328]**.

The researchers used female (NZB × NZW)F_1_ mice to study to effects of three estrogenic pesticides. They removed the ovaries of some of the animals to eliminate endogenous estrogen, while others were left intact. They then dosed the ovariectomized animals with the synthetic estrogen 17β-estradiol or with one of two doses of DDT, methoxychlor, or chlordecone. SLE can result in kidney failure due to glomerulonephritis, a deterioration of the glomerules that “filter” the blood, retaining nutrients and discarding wastes. So the team chose glomerulonephritis as an end point, and determined onset of the condition by measuring protein and blood urea nitrogen in the urine.

All three pesticides accelerated the onset of SLE. DDT and methoxychlor appeared to have roughly the same influence on SLE development as endogenous estrogen. The lower dose of methoxychlor tested—equaling approximately 1.2 milligrams per kilogram per day—produced kidney damage, even though it was fourfold lower than the no-observable-effect level used by the U.S. Environmental Protection Agency to calculate an oral reference dose for methoxychlor. This, the authors wrote, suggests that autoimmunity might be among the most sensitive measures of harm caused by methoxychlor.

Chlordecone produced the strongest response, so the team experimented further with lower doses of this compound. Chlordecone was commonly used in agriculture and some household products to kill ants and roaches in the 1960s and early 1970s. It was banned in the United States in the late 1970s, but it degrades slowly and still persists at some contaminated sites.

Examination of the kidney revealed a deterioration characteristic of the usual course of SLE, including the appearance of immune complexes of antibodies and target cells, even at the lowest chlordecone dose—a dose that would usually be considered a no-observable-effect level. Chlordecone also raised levels of antibodies to native, double-stranded DNA, another specific marker for SLE.

Despite evidence that all three pesticides accelerated the course of SLE, the chemicals did not increase uterine weight, which is a normal result of estrogen treatment. The authors speculate, therefore, that uterine hypertrophy might be a poor measure of the estrogenic effects of these pesticides, or that estrogenicity is not the mechanism that links pesticide exposure to SLE and renal failure.

Interestingly, the organochlorines caused about the same level of disease as the synthetic estrogen used as a positive control. However, because the mice studied almost always develop SLE, the research did not demonstrate that the organochlorines would cause SLE in animals that would not otherwise get the disease.

